# Dramatic response to alectinib in an *ALK* ‐positive LCNEC patient with a poor performance status: A case report

**DOI:** 10.1002/rcr2.817

**Published:** 2021-08-03

**Authors:** Kazuki Masuda, Masafumi Saiki, So Shimamura, Shuichiro Ide, Yoshinori Uchida, Yusuke Sogami, Hiroshi Ishihara, Fumi Ikeda, Kiyotaka Kugiyama

**Affiliations:** ^1^ Department of Internal Medicine II University of Yamanashi, Faculty of Medicine Chuo Japan; ^2^ Department of Internal Medicine Nirasaki City Hospital Nirasaki Japan

**Keywords:** alectinib, echinoderm microtubule‐associated protein‐like 4‐anaplastic lymphoma kinase, large cell neuroendocrine carcinoma, poor performance status

## Abstract

The echinoderm microtubule‐associated protein‐like 4 (*EML4*)–anaplastic lymphoma kinase (*ALK*) fusion gene, a driver mutation in lung carcinoma, is fairly common in lung adenocarcinoma but rare in large cell neuroendocrine carcinoma (LCNEC). Here we report a case of stage IV LCNEC positive for this fusion gene in a patient with a poor performance status (PS) who was effectively treated with alectinib. The patient was a 72‐year‐old non‐smoking man diagnosed as LCNEC with multiple metastases. Because of his poor PS, cytotoxic chemotherapy was not indicated, but he was later found to be positive for the *ALK* fusion gene and treated with alectinib as first‐line therapy. One month later, the tumour had shrunk remarkably, and the therapeutic effect was rated as a partial response. The PS also improved from 4 to 1. Investigating actionable driver mutations seems worth doing for advanced LCNEC, especially if the patient's PS is poor.

## Graphical abstract

We report a patient with LCNEC harbouring ALK rearrangement with a poor performance status, who showed a good response to alectinib as first‐line treatment. The patient's PS also improved, made him eligible for further chemotherapy when alectinib had failed.

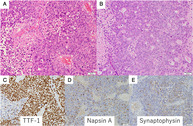

## INTRODUCTION

Large cell neuroendocrine carcinoma (LCNEC) is a rare type of non‐small cell lung carcinoma (NSCLC), but its clinical features are similar to those of small cell lung cancer (SCLC). Thus, stage IV LCNEC is generally treated with cytotoxic SCLC regimens,^1^, ^2^ although the optimal treatment has not been established due to its rarity.

The *EML4*–*ALK* fusion gene is found in approximately 3%–5% of NSCLC cases and is more commonly observed in adenocarcinomas.^3^ *ALK* fusion gene have been found occasionally in LCNEC as well,^4^, ^5^, ^6^, ^7^, ^8^, ^9^ but because their frequency is not known and LCNEC clinical features are SCLC‐like, they are not routinely investigated in clinical practice.

## CASE REPORT

**FIGURE 1:**
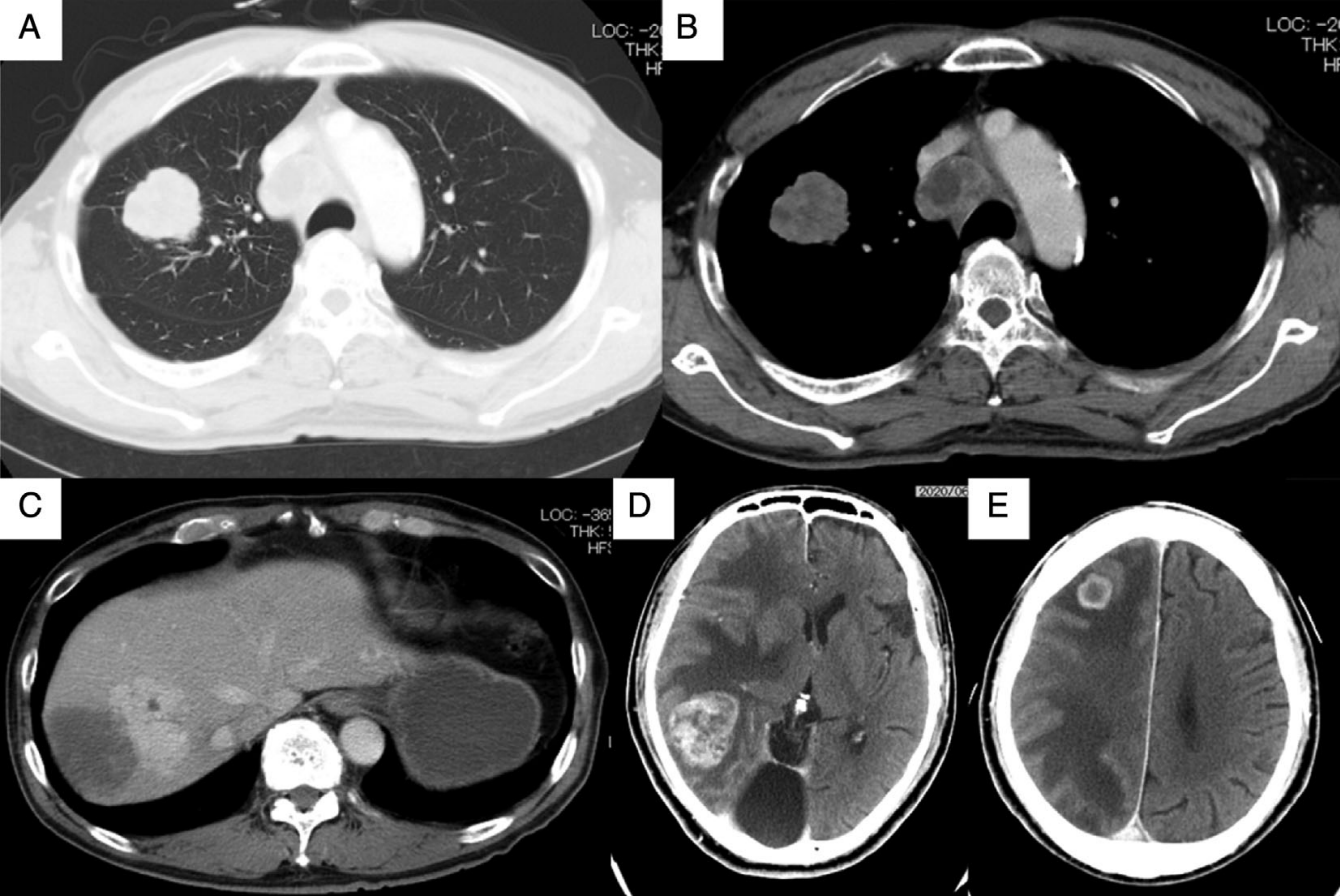
Systemic contrast‐enhanced computed tomography (CT) images before alectinib treatment. (A,B) Chest CT revealed a 38 mm mass in the right upper lobe and multiple mediastinal lymph node metastases. (C) Abdominal CT revealed liver metastases. (D,E) Head CT revealed multiple brain metastases in the right occipital and right parietal lobes

**FIGURE 2:**
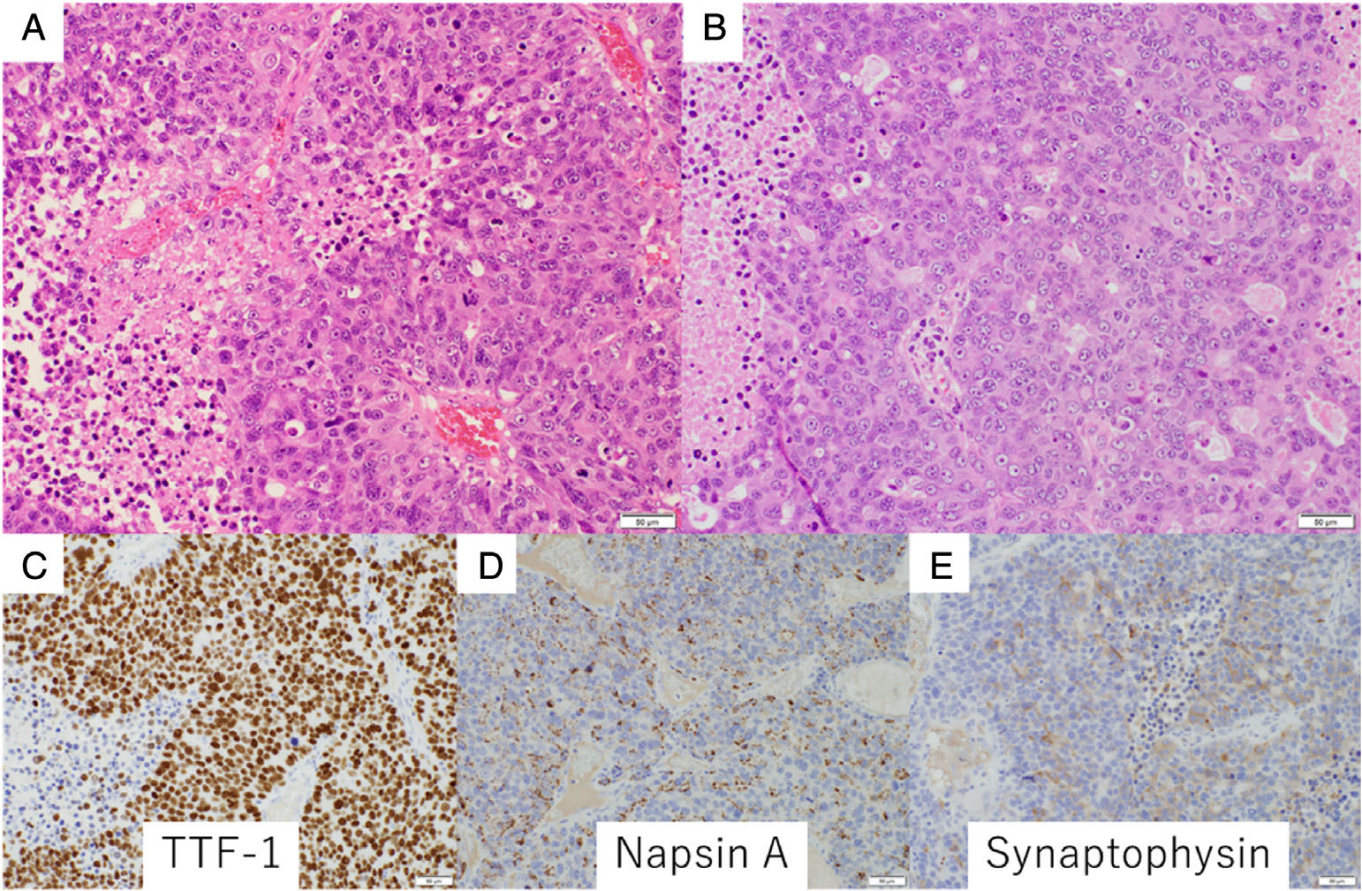
Histopathological features of the mass in the right occipital lobe. (A,B) Haematoxylin and eosin staining showed proliferation and necrosis of atypical cells with abundant cytoplasm and clear nucleoli. (C–E) Immunostaining indicated positivity for TTF‐1 (C), weak positivity for napsin A (D), and positivity for synaptophysin (E)

**FIGURE 3:**
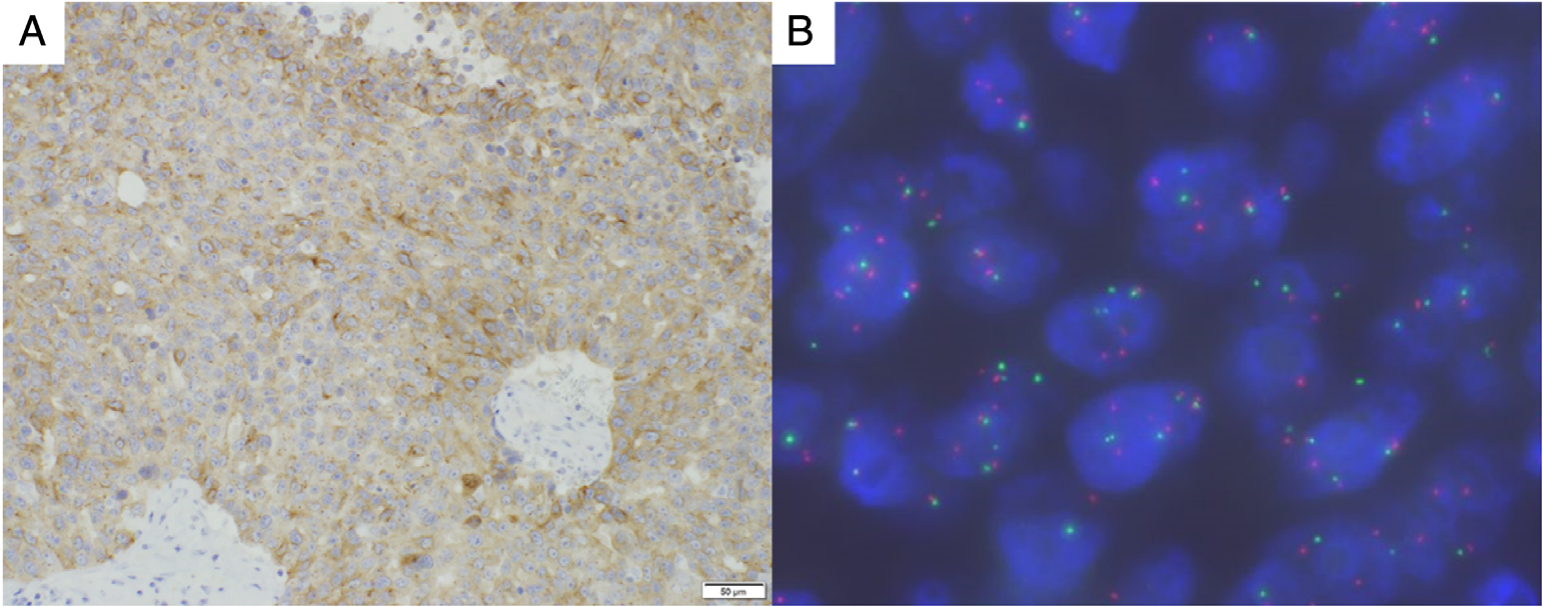
(A) Immunostaining indicated diffuse positivity for *ALK*. (B) *ALK* fluorescent in situ hybridization showed that 96% of tumour cells were positive for *ALK* rearrangement

**FIGURE 4:**
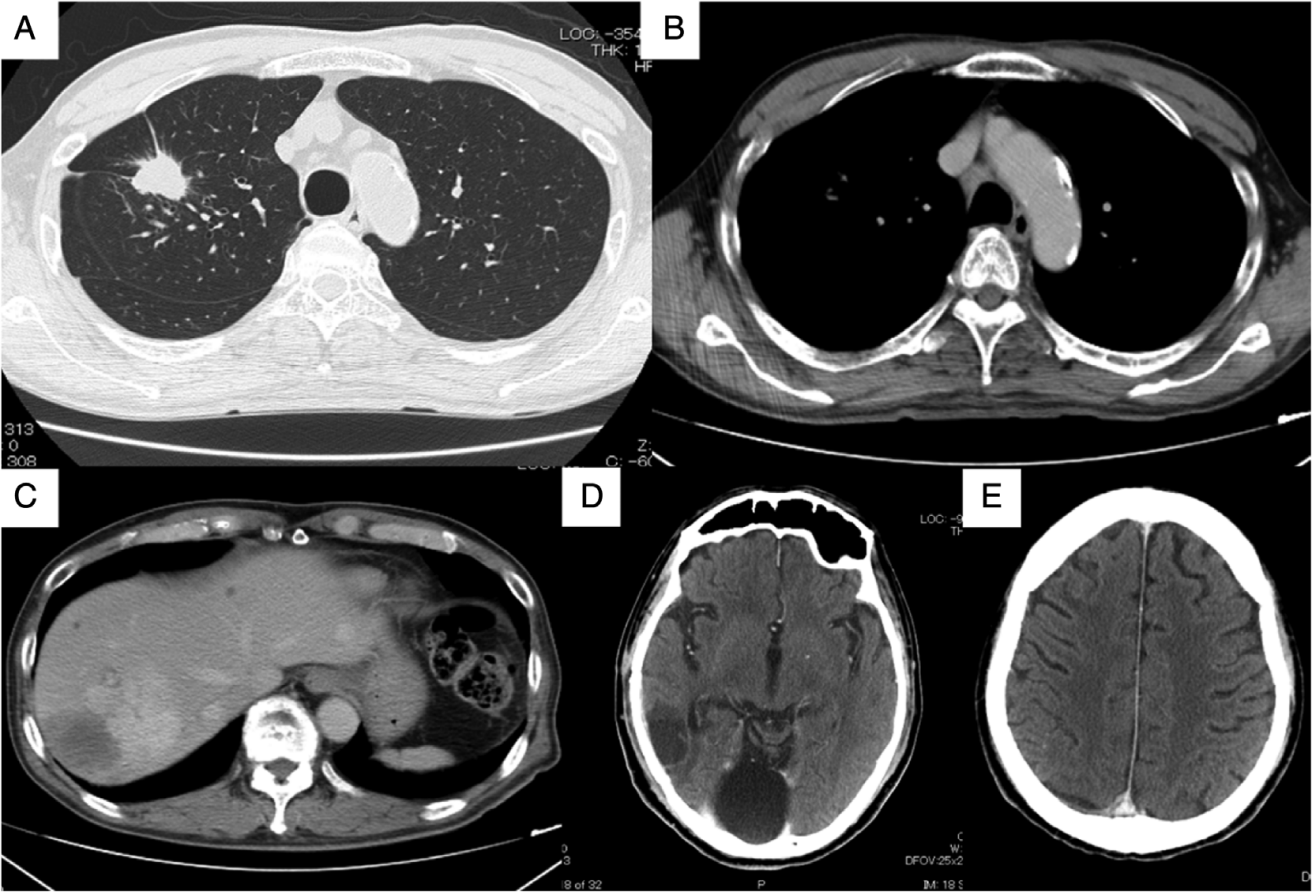
Systemic contrast‐enhanced computed tomography (CT) images at 1 month after starting alectinib. (A,B) Chest CT showed shrinkage of the lung carcinoma to 24 mm and of the metastatic lesions in the mediastinal lymph nodes. (C) Abdominal CT showed shrinkage of the liver metastases. (D,E) Head CT showed that the brain metastases in the right parietal lobe had disappeared completely. Brain metastases in the right occipital lobe were after surgical resection, so cannot be included in the evaluation of the therapeutic effect of alectinib

The patient was a 72‐year‐old non‐smoking man complicated with type 2 diabetes mellitus, chronic renal failure, hypertension and hyperuricaemia. He developed a gait disturbance in June 2020, followed by gradual development of disturbance of consciousness, when he was referred and admitted to our hospital. On admission, his consciousness was E3V4M6 by Glasgow Coma Scale, and his performance status (PS) was 4. Systemic contrast‐enhanced computed tomography (CT) showed a 38‐mm mass lesion in his right upper lobe and multiple metastases in his liver, left adrenal gland, mediastinal lymph nodes and brain (Figure 1A–E). Cerebral mass resection was performed, and pathological examination revealed proliferation and necrosis of atypical cells with abundant cytoplasm and clear nucleoli (Figure 2A,B). The tumour cells were positive for TTF‐1 (Figure 2C), weakly positive for Napsin A (Figure 2D) and positive for synaptophysin (Figure 2E) in immunohistochemical studies, resulting in the pathological diagnosis of LCNEC. Because of his poor PS, cytotoxic chemotherapy was not indicated and palliative care was initiated. Afterwards, *ALK* immunostaining proved to be diffusely positive, and *ALK* fluorescent *in situ* hybridization confirmed *ALK* rearrangement (Figure 3A,B). Accordingly, the *ALK* tyrosine kinase inhibitor (*ALK*‐TKI) alectinib was initiated as first‐line therapy. One month later, CT showed shrinkage of the lung lesion to 24 mm (Figure 4A) and of all distant metastases as well (Figure 4B–E), with the therapeutic effect being rated as a partial response. His PS also improved from 4 to 1 without any major adverse events. In 4 months of the treatment, CT showed new liver metastases, but his PS remains good and second‐line treatment is being considered with either cytotoxic chemotherapy or lorlatinib.

## DISCUSSION

Here, we report a patient with advanced LCNEC who was not indicated for cytotoxic chemotherapy due to his poor PS but was effectively treated with alectinib on the identification of *ALK* rearrangement. His PS also improved, making him eligible for further chemotherapy when alectinib had failed.

Alectinib is a second generation *ALK*‐TKI and reported to be more efficacious and less toxic compared with first generation *ALK*‐TKI such as crizotinib,^10^, ^11^ which makes alectinib more suitable for patients with poor PS. In fact, its effectiveness has been reported for *ALK*‐positive NSCLC patients with poor PS irrespective of whether they have brain metastasis or not.^12^

Because of its SCLC‐like feature, LCNEC of advanced stage is generally treated with cytotoxic SCLC regimens.^1^, ^2^ However, occasional *ALK*‐positive LCNEC cases and with good responsiveness to *ALK*‐TKI have been reported.^4^, ^5^, ^6^, ^7^, ^8^, ^9^ Although *ALK*‐TKI may be the only therapy for the patients with poor PS, the *ALK* fusion gene in the LCNEC patients is not routinely investigated, partly because its incidence is unknown. A recent case series reported that it was found in three out of 76 LCNECs (including one case with combined LCNEC and adenocarcinoma, 3.9%),^7^ which is as frequent as in lung adenocarcinoma.^3^ On the other hand, in the attempt to genomically classify LCNEC, Rehktman et al. found no *ALK* alteration in 45 LCNEC cases.^13^

In addition to the uncertain incidence, SCLC‐like features of LCNEC might also make clinicians withhold investigating the *ALK* fusion gene routinely. Recently, LCNEC was shown to be molecularly heterogeneous and can be classified into three subsets: SCLC‐like (characterized by RB1/TP53 co‐inactivation), adenocarcinoma‐like (characterized by frequent KRAS and/or STK11 mutations) and carcinoid‐like (characterized by MEN1 mutations and a low mutation burden), comprising 40%, 56% and 4% of 45 LCNEC cases, respectively.^13^ This molecular heterogeneity may explain the clinical diversity among LCNEC cases. It was also reported that weak positive immunostaining of Napsin A, a specific marker of primary lung adenocarcinoma, was observed in 15% of the adenocarcinoma‐like subset but not in other LCNEC subsets,^14^ indicating that Napsin A can be a histological marker of the adenocarcinoma‐like subset. Considering that druggable mutations such as the *ALK* fusion gene and epidermal growth factor receptor (*EGFR*) mutation are more common in adenocarcinoma, it is tempting to speculate that positive immunostaining for Napsin A in LCNEC patients, as we observed in this case, could imply the possible existence of these mutations.

According to the previous case reports,^4^, ^5^, ^6^, ^7^, ^8^, ^9^ and from our case also, clinical features of *ALK*‐positive LCNEC appear to be rather different from those of prototypical LCNEC. It has been described that most LCNEC patients are older males with a heavy smoking history,^15^, ^16^ but patients with *ALK*‐positive LCNECs were often younger and non‐ or light‐smoking females,^4^, ^5^, ^6^, ^7^, ^8^, ^9^ the features of which are commonly seen in *ALK*‐positive NSCLC patients.^3^

On the other hand, it should be noted that *EGFR* mutant‐positive LCNEC has been also reported several times in the past.^17^, ^18^ Therefore, if a patient with LCNEC has clinical traits similar to those of NSCLC harbouring driver mutation such as *ALK* fusion gene or histopathological features suggestive of an adenocarcinoma‐like subset such as positive Napsin A immunostaining, it might be more worth investigating driver mutations including *ALK* fusion gene. Further research is needed to identify the relationship between clinical and histological features and existence of driver mutations in LCNEC patients.

## CONFLICT OF INTEREST

None declared.

## ETHICS STATEMENT

Appropriate written informed consent was obtained for publication of this case report and accompanying images.

## AUTHOR CONTRIBUTIONS

Kazuki Masuda wrote the manuscript. So Shimamura, Shuichiro Ide, Yoshinori Uchida and Fumi Ikeda were involved in the interpretation of the data. Yusuke Sogami and Kiyotaka Kugiyama were involved in analysis of the data. Masafumi Saiki and Hiroshi Ishihara provided expertise and feedback. All authors drafted the article, revised it critically for important intellectual content and approved the final version to be submitted.
